# Effect of comprehensive rehabilitation on apnea hypopnea index in patients with obstructive sleep apnea: a protocol for randomized controlled trial

**DOI:** 10.1007/s11325-023-02982-2

**Published:** 2023-12-30

**Authors:** Mrudula Pawar, Prem Venkatesan, Satyanarayana Mysore, Guruprasad Bhat

**Affiliations:** 1 Department of Physiotherapy, Manipal College of Health Professions, Manipal Academy of Higher Education, Manipal, India; 2 Department of Pulmonology, Manipal Hospital, Bangalore, Karnataka India

**Keywords:** Obstructive sleep apnea, Myofunctional therapy, Breathing exercise, Daytime somnolence, Sleep quality

## Abstract

**Purpose:**

The aim of this study is to investigate the effect of comprehensive rehabilitation on apnea hypopnea index (AHI) in patients with obstructive sleep apnea (OSA).

**Methods:**

Patients diagnosed with OSA and meeting the eligibility criteria will be randomly allocated in the groups. The experimental group will receive comprehensive rehabilitation, and the control group will receive myofunctional therapy. CPAP will be continued by all the participants. Both the groups will receive the interventions for 12 weeks. The primary outcome measures are AHI and Epworth Sleepiness Scale (ESS), and secondary outcomes are Pittsburg Sleep Quality Index (PSQI), Oxygen Desaturation Index (ODI), Snoring Index (SI), Manual Assessment of Respiratory Motion (MARM), Breath Hold Test (BHT), and Self Evaluation of Breathing Questionnaire (SEBQ). The outcomes will be assessed at baseline and at the end of 12 weeks. A follow-up will be taken at the end of 24 weeks. Power analysis suggests that enrollment of 118 patients will required. Repeated measures ANOVA will be used to analyze the effect of the intervention.

**Conclusion:**

By performing this research, we may develop insights on a novel comprehensive approach for treatment of patients with OSA.

**Trial registration:**

CTRI/2023/10/058486.

## Introduction

Obstructive sleep apnea (OSA) presents with arousals from sleep at night due to collapse of oropharyngeal airway. OSA commonly results in excessive daytime sleepiness [1, 2]. Loud snoring, choking, and gasping during sleep are reported by the bed partners of these individuals with OSA [3]. The breathing pattern in OSA shows predominant thoracic breathing with a paradoxical motion of the abdomen [1, 4]. Patients with OSA tend to have oral and oro-nasal breathing in sleep [5].

The episodes of hypopnea and apnea during sleep are measured by polysomnography or home sleep testing with apnea hypopnea index (AHI) being a vital measure of the test [6]. Pharmacological management of OSA is not approved [7]. However, a recent systematic review [8] concluded that pharmacological treatment can be used as an adjunct therapy though it has only mild to moderate effect on OSA severity. Medications for OSA have potential negative effects. Hence, non-pharmacological management involving continuous positive airway pressure (CPAP) is considered to be the first line of treatment [9]. Evidence has shown the effectiveness of CPAP in decreasing the severity of OSA and improving sleep quality [10]. However, effects of CPAP may result in decreased adherence to treatment [2].

Myofunctional therapy serves as an adjuvant treatment for OSA. Myofunctional therapy involves isotonic and isometric oropharyngeal exercises, targeting the tongue and pharyngeal musculature [11]. A recent systematic review [12] has established the positive effects of myofunctional therapy in OSA. Another study, [13] showed the effectiveness of functional nasal breathing rehabilitation techniques on patients with nasal obstruction and mouth breathing. These patients reportedly decreased mouth breathing, reduced snoring, and improved sleep [13]. Therefore further studies are needed on this technique in patients with impaired sleep.

To date, publications have reported on the usefulness of CPAP and myofunctional therapy as treatment approaches for OSA. Thus, a randomized controlled trial to assess the effectiveness of comprehensive rehabilitation for OSA is justifiable. The aim of the study is to investigate the effect of comprehensive rehabilitation on AHI in patients with OSA. The primary objectives of the study are to determine the effect of comprehensive rehabilitation on AHI and excessive day time sleepiness. The secondary objectives are to determine effects on oxygen desaturation, snoring, sleep quality, and on the components of breathing dysfunction.

## Method

### Study design

**Fig. 1:**
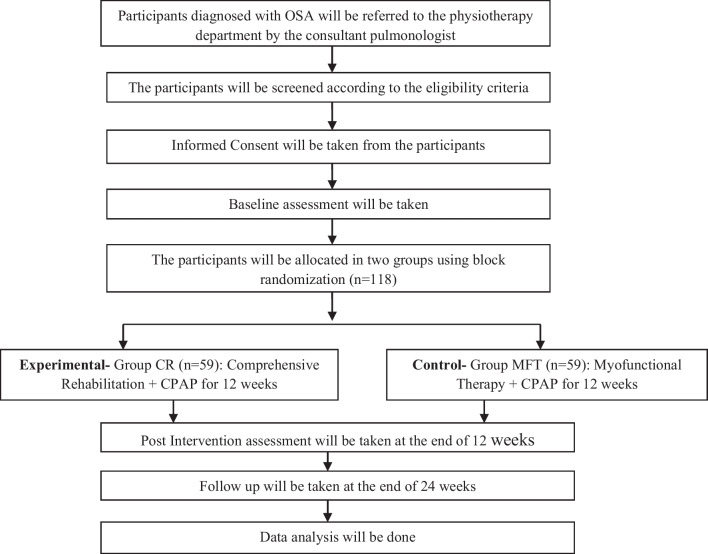
Study flow chart

The study design for the current protocol is an “assessor-blinded parallel group randomized controlled trial.” The trial will use the “Consolidated Standards of Reporting Trials (CONSORT) guidelines” [14]. All outcomes will be assessed at baseline and at the end of 12 weeks. Additionally, a follow-up will occur at the end of 24 weeks with assessment of daytime sleepiness, sleep quality, and breathing pattern dysfunction. The study flow is shown in Fig. 1. Reporting of the protocol is done according to the “Standard Protocol Items: Recommendations for Intervention Trials (SPIRIT) guidelines” [15]. The present study is approved by Institutional Research Committee (IRC) of Manipal Academy of Higher Education. Scientific Committee approval and ethical clearance to conduct the study is obtained from Manipal Hospital, Bangalore. The study is registered in Clinical Trials Registry–India (CTRI) (registration number—CTRI/2023/10/058486).

### Participant recruitment, sample size, and eligibility criteria

According to the American Academy of Sleep Medicine (AASM) criteria, participants will be diagnosed with OSA by the consultant pulmonologist [16]. The recruitment of the participants will take place at Manipal Hospital, Bangalore, from October, 2023, to February, 2026.

The sample size was calculated at 5% level of significance with 80% power and an effect size of 0.30. The drop-out rate (20%) and the design effect (1.5) were also incorporated. Thus, the sample derived was 116.25 and 118 will be enrolled, with 59 in each group. The eligibility criteria are outlined in Table 1.

**Table 1:** Eligibility criteria

Inclusion criteria	Exclusion criteria
Mild and moderate OSA (AHI ≥ 5 to ≤ 30)	Obstructive nasal diseases
Age 25 to 65 years	Craniofacial malformations
Either gender	Comorbid sleep disorders such as insomnia, restless leg syndrome
BMI less than 40 kg/m^2^	Pregnant women
	History of pharyngeal surgery
	History of congestive heart failure, cardiomyopathy, chronic obstructive pulmonary disease, and liver cirrhosis
	History of neuromuscular disorders such as myasthenia gravis, motor neuron disease, and demyelinating diseases
	History of stroke
	Psychiatric illness
	Consumption of alcoholic beverages (more than 3 oz/day or more than 2 alcoholic beverages/day)
	Periodontal disease (gingivitis]
	Temporomandibular joint dysfunction

### Randomization, allocation, and blinding

The randomization will be done using computer-generated block randomization. Allocation will be done using sealed envelopes. The allocation sequence will be kept safely in “sequentially numbered opaque sealed envelopes.” The eligible participants will be informed about the trial process by the principal investigator and an informed consent will be sought. A blinded assessor will collect outcome measures at baseline, at the end of 12 weeks, and at the end of 24 weeks. The treatment will be given to the participant by the therapist according to that mentioned in the sealed envelope.

### Intervention

#### CR group: comprehensive rehabilitation + CPAP

The comprehensive rehabilitation for OSA will include 3 components: functional nasal breathing rehabilitation, oropharyngeal exercises (myofunctional therapy], and diaphragmatic breathing exercise.

#### Functional nasal breathing rehabilitation [13]

This technique will include three exercises, including breath hold, humming and its variation, and nose open smile. A rest interval of 2 min will be given between each exercise. All exercises will repeated 5 times.

#### Oropharyngeal exercises [17]

These maneuvers include soft palate exercises, tongue exercises, facial exercises, and stomatognathic functions. Each exercise will be repeated 5 times. Isometric exercise of the muscles will be performed with 10-s hold. For stomatognathic functions, the subject will be advised to perform balloon blowing exercises and to chew bilaterally while having food.

#### Diaphragmatic breathing exercise [18]

This exercise will be performed 5 times by the subject.

This protocol will be followed 3 times per day for 5 days per week for 12 weeks. The treatment will be supervised for the 1st week. From the 2nd week, weekly supervised sessions will be held with the participants. A weekly exercise diary will be provided to the patients to document their adherence to treatment.

#### MFT group: myofunctional therapy + CPAP

The myofunctional therapy will include the oropharyngeal exercises of soft palate, tongue, and face as outlined above. Myofunctional therapy will be performed 3 times per day, 5 days per week for 12 weeks.

CPAP treatment will be given to the subjects daily in both the groups for 12 weeks. The CPAP prescription will be followed as directed by the pulmonologist. CPAP data will be noted to record compliance. Compliance will be defined as use of the CPAP for ≥ 4 h per night for at least 70% of nights in the 12 weeks will be followed [19].

### Outcomes

AHI and ESS will be the primary outcomes. Secondary outcomes will be Oxygen Desaturation Index, Snoring Index, Pittsburgh Sleep Quality Index, Manual Assessment Of Respiratory Motion, Breath Hold Test, and Self Evaluation of Breathing Questionnaire.

#### Home sleep apnea testing (HSAT)

Home sleep apnea testing will be used to diagnose and measure severity of OSA as recommended by the American Sleep Disorders Association (ASDA) [20, 21]. According to Sleep, Cardiovascular, Oximetry, Position, Effort, and Respiratory (SCOPER) parameters, the HSAT that will be used can be denoted as denoted as S_0_, C_3_, O_1X_, P_2_, E_1_, and R_2_ [22]. HSAT has a high sensitivity (82%), specificity (88%), and positive predictive value of (89%) [21, 23].

The parameters measured are as follows:

- Apnea Hypopnea Index (AHI)—the average number of events of apnea and hypopnea per hour during sleep. AHI is used to determine the severity of OSA as mild (AHI 5 up to 15), moderate (AHI 15 up to 30), and severe (AHI 30 or more).
- Oxygen Desaturation Index (ODI)—the average number of episodes of oxygen desaturation per hour.
- Snoring Index (SI)—the measure of number of snoring events per hour during sleep.

#### Epworth Sleepiness Scale (ESS)

The ESS is a self-administered questionnaire used to measure the level of daytime sleepiness of a subject. The ESS consists of 8 items which are rated on a scale of 0 (no chance of dozing) to 3 (high chance of dozing) yielding a total score ranging from 0 to 24. The scale has test–retest reliability (Pearson’s correlation coefficient, *r* = 0.82), high degree of internal consistency (Cronbach’s *α* = 0.88) and validity [24].

#### Pittsburgh Sleep Quality Index (PSQI)

The PSQI is used to assess the quality of sleep and disturbances during sleep. The PSQI is a reliable and valid self-rated questionnaire. PSQI consists of 9 items that are divided into 7 components. For scoring, these 7 components are each scored from 0 (no difficulty) to 3 (severe difficulty) summing to produce a global PSQI score ranging from 0 to 21, with higher scores indicating declining sleep quality. The scale has a high degree of internal consistency (Cronbach’s *α* = 0.83), good test–retest reliability (Pearson’s correlation coefficient, *r* = 0.85), and validity [25].

#### Manual Assessment of Respiratory Motion (MARM)

The MARM is reported to be a valid and reliable tool for clinical and research purposes [26], used to evaluate and quantify breathing patterns with respect to the ribcage and abdominal movement. The volume, balance, and percentage of ribcage motion will be recorded.

#### Breath Hold Test (BHT)

The Breath Hold Test is a reliable and valid method to assess biochemical component of breathing dysfunction [27, 28]. Three maximal breath holds will be performed at 3-min rest intervals each. The mean of three readings will be used to determine the results.

#### Self Evaluation of Breathing Questionnaire (SEBQ)

The SEBQ is a self-reported outcome measure to determine breathing related symptoms. The SEBQ has 25 items, each scored from 0 (never/not true at all) to 3 (frequently/very true). A total score greater than 11 shows that the individual has a breathing problem. The SEBQ has a high test–retest reliability [intraclass correlation coefficient (3, 1) = 0.89; 95% CI 0.85–0.92], internal consistency (Cronbach’s *a* = 0.93), and validity [29].

### Data monitoring and management

Proper execution of the research and its progress’ responsibility will be the responsibility of the principal investigator. The data monitoring and safety committee (DMSC) will check for the accuracy of the data and participants’ safety. The research progress will be updated with the DMSC through regular meetings every 6 months. Participants will be informed to report any adverse event to the investigator. Collected data will be stored in secured cabinets. Microsoft excel will be used to store the participants’ deidentified data. The number of participants not adherent to the study, withdrawals, and those who are lost to follow-up will be noted along with the reasons.

## Results

Statistician will be provided deidentified data, and analysis will be performed. Descriptive statistics (frequency tables and percentages for categorical variables and for computation of prevalence) will be used.

Mean/median and standard deviation/inter-quartile range will be analyzed after exploring skewness for the quantitative variables. Repeated-measures ANOVA or its non-parametric variants will be used after exploring the model assumptions to assess the effectiveness of the intervention. Correlation analysis may be done to explore associations between the outcomes.

Regression models may be used for adjustment of covariates to account the confounding factors and for unbiased treatment effect. Intention to treat analysis will be used to manage missing data. Inferential statistics will be used for exploratory data analysis.

## Discussion

The current study includes functional nasal breathing rehabilitation along with myofunctional therapy and diaphragmatic breathing as a comprehensive management protocol for patients with OSA. Myofunctional therapy as a part of conventional treatment for OSA, in addition to CPAP, has reported to have a positive effect on the tone of oropharyngeal muscles and other soft tissues of the upper airway [11]. It may be hypothesized from the preceding research that functional nasal rehabilitation and diaphragmatic breathing exercise may be beneficial for patients with OSA [13, 18]. 

A limitation of this study as designed is that it will be performed in a specific region, and the data cannot be generalized to other populations and settings.

## Data Availability

The present study has no data.
